# Growth arrest specific gene 2 in tilapia (*Oreochromis niloticus*): molecular characterization and functional analysis under low-temperature stress

**DOI:** 10.1186/s12867-017-0095-y

**Published:** 2017-07-17

**Authors:** ChangGeng Yang, Fan Wu, Xing Lu, Ming Jiang, Wei Liu, Lijuan Yu, Juan Tian, Hua Wen

**Affiliations:** Key Laboratory of Freshwater Biodiversity Conservation and Utilization of Ministry of Agriculture, Yangtze River Fisheries Research Institute, Chinese Academy of Fishery Sciences, Wuhan, 430223 China

**Keywords:** *Oreochromis niloticus*, Growth arrest specific gene 2, Functional characterization, Low-temperature stress

## Abstract

**Background:**

Growth arrest specific 2 (*gas2*) gene is a component of the microfilament system that plays a major role in the cell cycle, regulation of microfilaments, and cell morphology during apoptotic processes. However, little information is available on fish *gas2*. In this study, the tilapia (*Oreochromis niloticus*) *gas2* gene was cloned and characterized for the first time.

**Results:**

The open reading frame was 1020 bp, encoding 340 amino acids; the 5′-untranslated region (UTR) was 140 bp and the 3′-UTR was 70 bp, with a poly (A) tail. The highest promoter activity occurred in the regulatory region (–3000 to –2400 bp). The Gas2-GFP fusion protein was distributed within the cytoplasm. Quantitative reverse transcription-polymerase chain reaction and western blot analyses revealed that *gas2* gene expression levels in the liver, muscle, and brain were clearly affected by low temperature stress. The results of *gas2* RNAi showed decreased expression of the *gas2* and P53 genes.

**Conclusion:**

These results suggest that the tilapia *gas2* gene may be involved in low temperature stress-induced apoptosis.

**Electronic supplementary material:**

The online version of this article (doi:10.1186/s12867-017-0095-y) contains supplementary material, which is available to authorized users.

## Background

Growth and reproduction in fish are closely related to water temperature, light, gas pressure, and other climatic factors. Among them, ambient temperature is the most important factor affecting growth and development of fish [1–3]. In recent years, more and more attention has been paid to the molecular changes in fish responding to a low temperature environment [4–6]. Tilapia is a widely cultured warm water fish worldwide, with poor low temperature tolerance, and a growth temperature range of 16–38 °C. Therefore, culture is clearly subject to climate and temperature restrictions [7]. Studying the physiology, biochemistry, and molecular mechanisms of tilapia under low temperature stress will help understand the effects of low temperature on fish and provide a theoretical basis for the tilapia breeding industry.

Transcriptome and digital gene expression (DGE) analyses were used in our previous study to analyze the gene expression changes in the liver of tilapia under low temperature stress [8]. As results, many differentially expressed genes involved in growth, development, immunity, apoptosis, and other physiological processes were detected. Among them, a gene that plays an important role in apoptosis, called growth arrest specific 2 gene (*gas2*), was identified. *Gas* genes are upregulated during serum starvation or cell contact inhibition in vitro [9, 10]. The protein encoded by *gas2* is a component of the microfilament system. It is highly conserved during evolution and plays a major role in the cell cycle, regulation of microfilaments, and cell morphology during apoptotic processes, and regulation of calpain activity [11–14]. In this study, we cloned the tilapia *gas2* gene and analyzed structure and active region of its promoter, and subcellular localization of the protein. The *gas2* gene expression profile was analyzed in tilapia subjected to low-temperature stress. The relative changes observed will provide insight into the effect of low-temperature stress on tilapia.

## Methods

### Experimental fish

Tilapia was purchased from a fish hatchery in Guangxi Province, China and was transported to the experimental laboratory at the Yangtze River Fisheries Research Institute (Wuhan, Hubei Province, China). Their initial body weight was 140.0 ± 10 g. The fish were initially held for 2 weeks at 28 °C before commencing the experiments. Then, water temperature was increased to 30 °C for 1 week before they were subjected to a decrease in water temperature from 30 to 10 °C, which is near the lethal minimum [15], at a cooling at a rate of 1 °C/day. Dissolved oxygen was maintained at >5 mg/L with an air compressor, water pH was 7.2–7.5, and NH_3_–N was 0.26 ± 0.10 mg/L.

### Sample preparation

Nine tissues (brain, gill, skin, muscle, heart, liver, eye, spleen, and intestines) were collected from each of three fish held at 30 °C. Three fish each from temperatures of 30, 25, 20, 15, and 10 °C were anesthetized, and samples of liver, brain, and muscle were collected, frozen in liquid nitrogen, and stored at −80 °C until use. Total RNA was extracted from these samples using Trizol reagent (Invitrogen, Carlsbad, CA, USA), according to the manufacturer’s instructions. Liver, brain, and muscle tissues were ground in liquid nitrogen to extract total protein. Then, 300 µL of ice-cold lysis buffer (150 mM NaCl, 1.0% NP-40, 0.5% sodium deoxycholate, 0.1% sodium dodecyl sulfate, 50 mM Tris–HCl, pH 8.0, and protease inhibitors) were added to 5 mg tissue samples and maintained for 2 h at 4 °C with constant agitation. After a 20 min centrifugation at 12,000 rpm in a microcentrifuge at 4 °C, the tubes were placed on ice, the supernatant was aspirated to a fresh tube on ice, and the pellet was discarded.

### Cloning the tilapia *gas2* gene

Based on the DGE-tag sequences obtained and the reference tilapia genome data (https://www.ncbi.nlm.nih.gov/genome/197?genome_assembly_id=293496), four specific primers (Additional file 1: Table S1) were designed to perform the 5′ and 3′ rapid amplification of cDNA ends (RACE) procedure on this gene using the BD SMART™ RACE cDNA amplification kit (BD Biosciences/Clontech, Palo Alto, CA, USA) following the manufacturer’s instructions.

Based on the cDNA sequences obtained and the reference tilapia genome data, two primers were designed to acquire the DNA promoter sequences (Additional file 1: Table S1).

### Sequence and phylogenetic analyses

The tilapia *gas2* motif was scanned using PROSITE (http://prosite.expasy.org/). The deduced amino acid sequence of tilapia *gas2* was submitted to the BLAST program (http://blast.www.ncbi.nlm.nih.gov) to search for counterpart sequences. Multiple sequence alignments were performed with the ClustalX 1.83 program. Then, an unrooted phylogenetic tree was constructed using the neighbor-joining algorithm in the MEGA 5.05 program, based on the sequence alignments and other Gas2 genes. The phylogenetic tree was tested for reliability by 1000 bootstrap replications. Putative transcription factor binding site motifs were detected by MatInspector software.

### Construction of a luciferase-reporter gene vector for the tilapia *gas2* gene promoter and pEGFP-N3-GAS2 gene expression vector

A promoter–deletion experiment was designed to monitor promoter activity of the 5′-flanking region. DNA fragments with a series of nested deletions were generated, and five DNA fragments were inserted into the pGL4.10 vector and named pGL4-1 (−3000 to 0 bp), pGL4-2 (−2400 to 0 bp), pGL4-3 (−1800 to 0 bp), pGL4-4 (−1200 to 0 bp), and pGL4-5 (−600 to 0 bp). pGL4.51 vector (Promega, E132A) with a CMV promoter was used as a reference (positive control), while pGL4.10 vector (Promega, E665A) without promoter was used as negative control.

The pEGFP-N3 expression plasmid was purchased from Invitrogen. The tilapia *gas2* open reading frame (ORF) was amplified by polymerase chain reaction (PCR), cloned into the pEGFP-N3 with the GAS-N-S1 and GAS-N-A1 primers (Additional file 1: Table S1), and named pEGFP-N3-GAS2.

### Construction of short hairpin RNA (shRNA) expression vectors

Three shRNA sequences were designed based on the *gas2* gene sequences. Three shRNA-expressing plasmids specifically targeting *gas2* (called shG1, shG2, and shG3) were constructed by GenePharma Corp. (Shanghai, China) using the pGPU6/Neo vector. Scrambled shRNA was used as a negative control. The shRNA sequences are shown in Additional file 2: Table S2.

### Cell culture, transient transfection, and detection of luciferase activity

The CHO-K1 cell line was purchased from the Institute of Biochemistry and Cell Biology, Shanghai Institutes for Biological Sciences, Chinese Academy of Sciences (Shanghai, China). CHO-K1 cells were cultured in F-12K medium containing 10% fetal bovine serum, 100 U/mL penicillin, and 100 U/mL streptomycin at 28 °C in 5% CO_2_. The cells were seeded in 96-well plates for 24 h before transfection. When the cells were 90% confluent, an equivalent quantity of plasmid was transfected into the CHO-K1 cells following the transfection reagent instructions (Lipofectamine™ 2000; Invitrogen).

### Detection of luciferase activity

The cells were harvested after 48 h of transfection, and luciferase activity was detected on Molecular Device 5 using a Luciferase Reporter Gene Assay Kit (Beyotime, Shanghai, China), in accordance with the manufacturer’s instructions. Three replicates were carried out for each sample.

### Culture of the tilapia brain cell line (TBC), sub-cellular localization, and tilapia *gas2* RNAi

The TBC tilapia brain cell line (construction by the Yangtze River Fisheries Research Institute) was maintained in L15 medium (Hyclone, Logan, UT, USA) containing 20% fetal calf serum (JIBC, USA) and antibiotics. The flasks/plates were seeded at 50% confluency before transfection and were transfected into 6-well plates at constant numbers. The cells were maintained in medium without fetal calf serum or antibiotics prior to transfection. When the cells grew to 90% confluence, an equivalent quantity of plasmids (pEGFP-N3-GAS2 or shRNA) was transfected into the TBC cells following the transfection reagent instructions (Lipofectamine™ 2000, Invitrogen). The cells were stained for sub-cellular localization 24 h later with 1 mg/mL DAPI for 1 h away from light. All samples were examined under a Leica SP8 confocal laser scanning microscope (CLSM; Leica Microsystems,Bannockburn, IL, USA). The cells were harvested to carry on quantitative reverse transcription-PCR (qRT-PCR) for RNAi 48 h later.

### Quantitative RT-PCR analysis

qRT-PCR was conducted to determine the *gas2* mRNA tissue distribution, the mRNA expression pattern under low-temperature stress, and expression of the P53 gene (Accession Number: XM_005463838). Expression of β-actin was used as the internal control. Total RNA was prepared with TRIzol reagent. cDNA was synthesized from each RNA sample (500 ng) using a PrimeScript^®^ RT reagent kit (Takara Bio, Shiga, Japan), following the manufacturer’s recommendations. qRT-PCR was conducted on an Applied Biosystems 7500 Real-Time PCR System with SYBR^®^ Premix Ex Taq™ (Takara Bio). The primer pair GAS-R-S1 and GAS-R-A1, P53-R-S1 and P53-R-A1, and Actin-S1 and Actin-A1 was used to amplify the *gas2*, P53, and β-actin fragments, respectively (Additional file 1: Table S1).

Real-time PCR was carried out with 1 μL cDNA sample, 10 μL SYBR^®^ Premix Ex Taq™, 0.4 μL ROX Reference Dye II, 0.4 μL PCR forward/reverse primers (10 mM), and 7.8 μL nuclease-free water. The thermocycling conditions for the reaction were as follows: 95 °C for 30 s, followed by 40 cycles consisting of 95 °C for 5 s, and 60 °C for 34 s. The reactions were carried out with three duplicates of each sample. The ratio changes in the target genes relative to the control gene (β-actin) were determined by the 2^−△△CT^ method.

### Recombinant expression and identification and preparation of the fusion protein antibody

The tilapia *gas2* coding sequence was amplified using the GAS-P-S1 and GAS-P-A1 primers and sub-cloned into pET-30a to construct pET-30a-Gas. Then, expression of the His-tagged fusion protein was induced with pET-30a-Gas using 0.5 mM isopropyl-b-D-thiogalactopyranoside at 37 °C for 3–4 h. A Ni-NTA column was used to purify the tilapia Gas2 fusion protein, followed by sodium dodecyl sulfate–polyacrylamide gel electrophoresis (SDS-PAGE) and a western blot assay (with anti-His antibody) to detect the recombinant tilapia Gas2 protein. Anti-Gas2 from rabbit was prepared by China Wuhan ABclonal Biotech Co., Ltd. (Wuhan, China).

### SDS-PAGE and western blot assay

Protein concentrations were determined using the BCA method, separated on a 12% polyacrylamide gel under reducing conditions, and transferred to a polyvinylidene difluoride membrane. The membranes were blocked in blocking buffer (TBST, 5% skimmed milk in TBS containing 0.05% Tween-20) for 1 h and incubated overnight at 4 °C with primary rabbit antibodies against Gas2 or β-actin (1:1000; ABclonal) in TBST containing 1% skimmed milk. After washing with TBST (3 × 15 min), the membranes were incubated with goat anti-rabbit horseradish peroxidase-conjugated IgG (1:1000; Tiangen) for 1 h at room temperature. The reactive protein bands on the membrane were visualized using enhanced chemiluminescent reagents (Tiangen) and exposed in a darkroom. The expression intensities of the Gas2-specific bands were normalized against the β-actin bands.

### Statistical analysis

One-way analysis of variance (ANOVA) was used to analyze the log-transformed Ct values. When ANOVA identified differences among groups, Tukey’s multiple-comparisons test (SAS Institute, Cary, NC, USA) was conducted to examine the differences among the treatments and values. A *p* value <0.05 was considered significant.

## Results

### Cloning and sequence analysis of the tilapia *gas2*

**Fig. 1:**
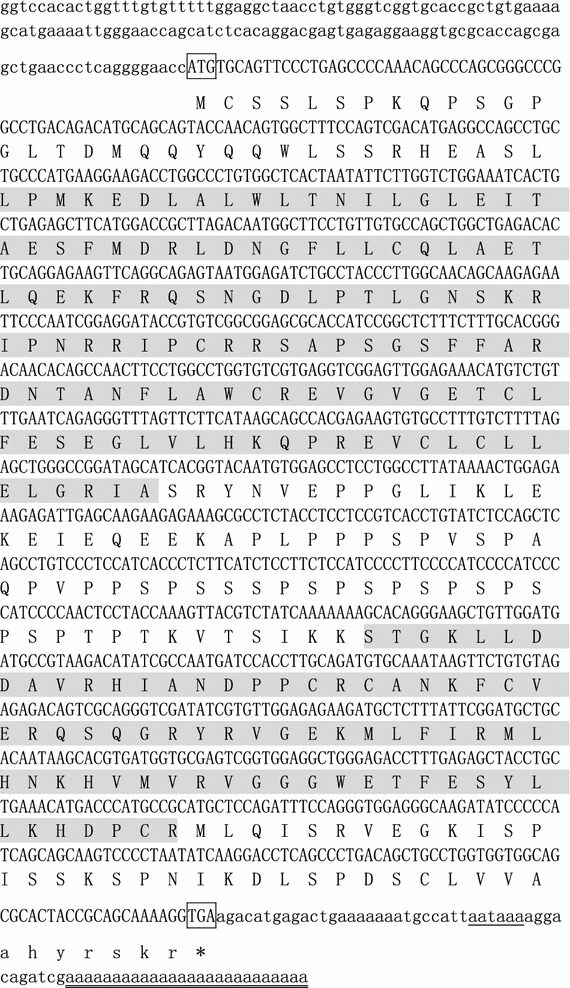
The cDNA and predicted protein sequence of tilapia Gas2. The open reading frame sequences are shown in *capital letters*. The start and stop codons are marked by *box*. The AATAAA box is *underlined*, and the poly(A) region is *double-underlined*. The calponin homology (CH) domain (pos.: 34–159) and Gas2-related (GAR) domain (pos.: 227–300) were in *shadow*, respectively

**Fig. 2:**
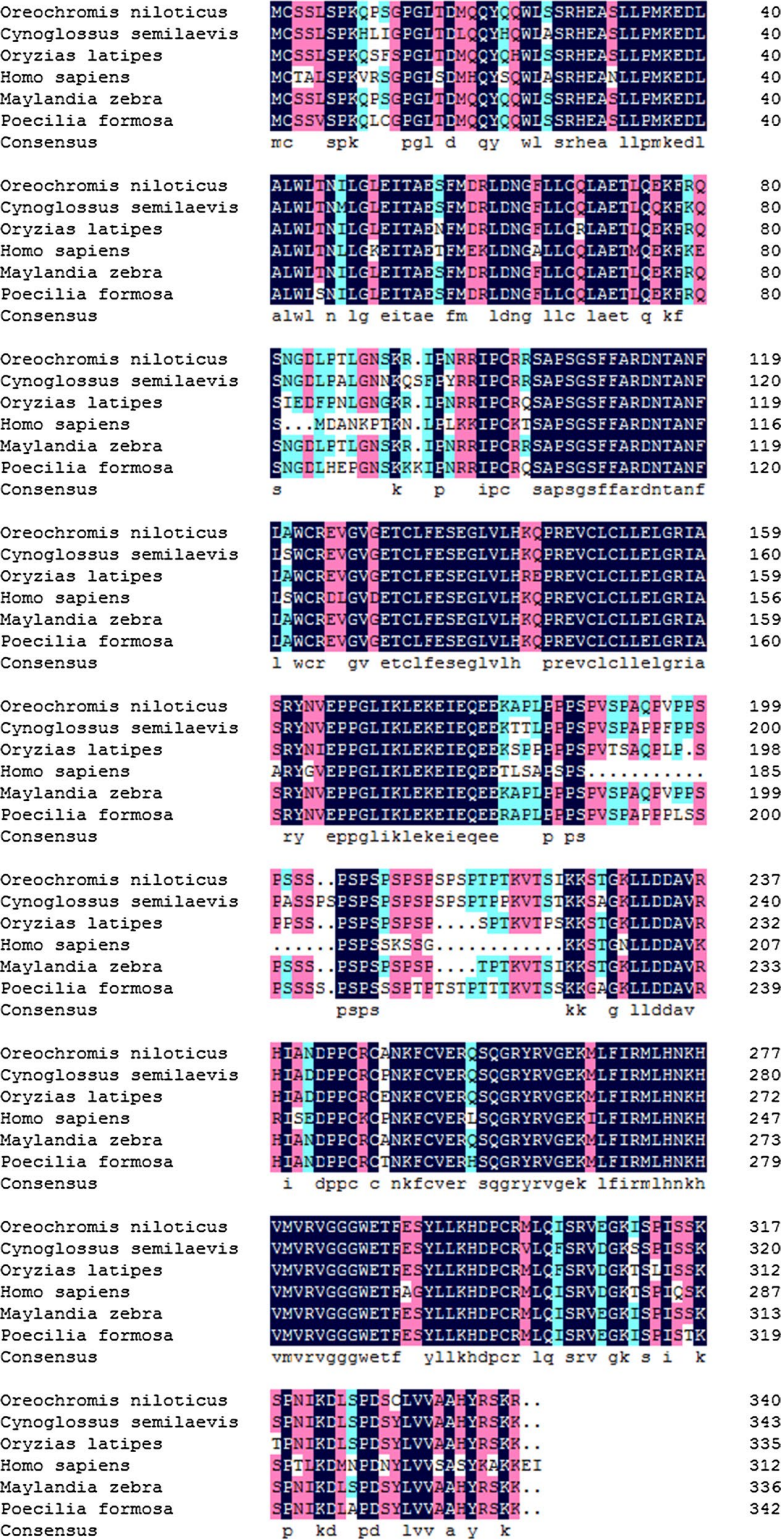
Multiple alignment of deduced amino acid sequences of Gas2 from *Cynoglossus semilaevis* (XP_008310260), *Oryzias latipes* (XP_004069628), *Homo sapiens* sequence (NP_005247), *Maylandia zebra* (XP_004575167), *Poecilia formosa* (XP_007578649)

We acquired the 1020-bp ORF of the tilapia *gas2* gene, encoding 340 amino acids based on the DGE-tag sequence. The molecular weight of the protein was determined to be 37.7 kDa, and the isoelectric point (pI) was 8.67. The 5′-UTR was 140 bp and the 3′-UTR was 70 bp with a poly (A) tail, according to 5′ and 3′ RACE cDNA amplification (Fig. 1). The sequence was submitted to Genbank (No. KY882138). The protein was identified as *gas2* and the calponin homology (CH) domain and *gas2*-related (GAR) domain consisted of 126 and 74 amino acid residues, respectively, according to a PROSITE analysis (Fig. 1). The closest homology was detected by multiple alignments of *gas2* deduced amino acid sequences with those of other species (Fig. 2).

### Phylogenetic analysis of tilapia *gas2*

A phylogenetic tree for the tilapia Gas2 protein and the other known Gas2 proteins was constructed by the neighbor-joining method. The list of species included is shown in Additional file 3: Figure S3. On the phylogenetic tree, tilapia Gas2 was distantly related with *Drosophila bipectinata* Gas2. And the tilapia Gas2 protein shared the closest relationship with *Neolamprologus brichardi* (Additional file 4: Figure S1).

### Cloning and promoter analysis of the tilapia *gas2* 5′-flanking region

**Fig. 3:**
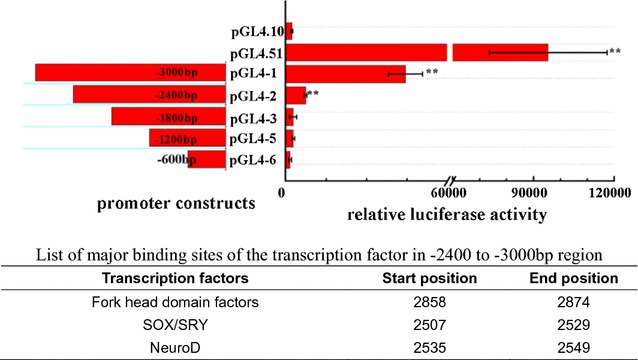
Results of 5 fragments analyzed with dual-luciferase reporter system. *pGL4-1–5* the activity of 1–5 in pGL4.10 vector, respectively, *pGL4.10* negative control, *pGL4.51* positive control. Significant differences are indicated with *two asterisks* at *p* < 0.01

3000 bp promoter sequence was cloned using a PCR method. As shown in Fig. 3, relative luciferase activity was detected from CHO-K1 cells transfected with the luciferase reporting assay vector. The luciferase activities from pGL4.51 (positive control), pGL4-1 (−3000 to 0 bp), and pGL4-2 (−2400 to 0 bp) were significantly higher than those in the negative control. Analysis of the promoter sequence in the region (−2400 to −3000 bp region) revealed that the main motifs of the sequence, e.g., Fox gene family, NeuroD, SOX/SRY and so on. (Fig. 3).

### Tilapia *gas2* expression profile

**Fig. 4:**
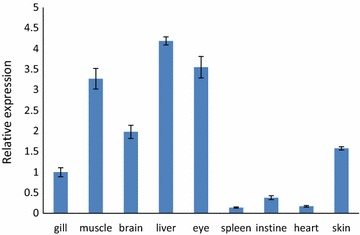
The quantitative RT-PCR analysis of *gas2* expression in tilapia different tissues in 30 °C. The β-actin gene was used as an internal control to calibrate the cDNA template for all the samples. Data are expressed as the mean ± SD (n = 3)

**Fig. 5:**
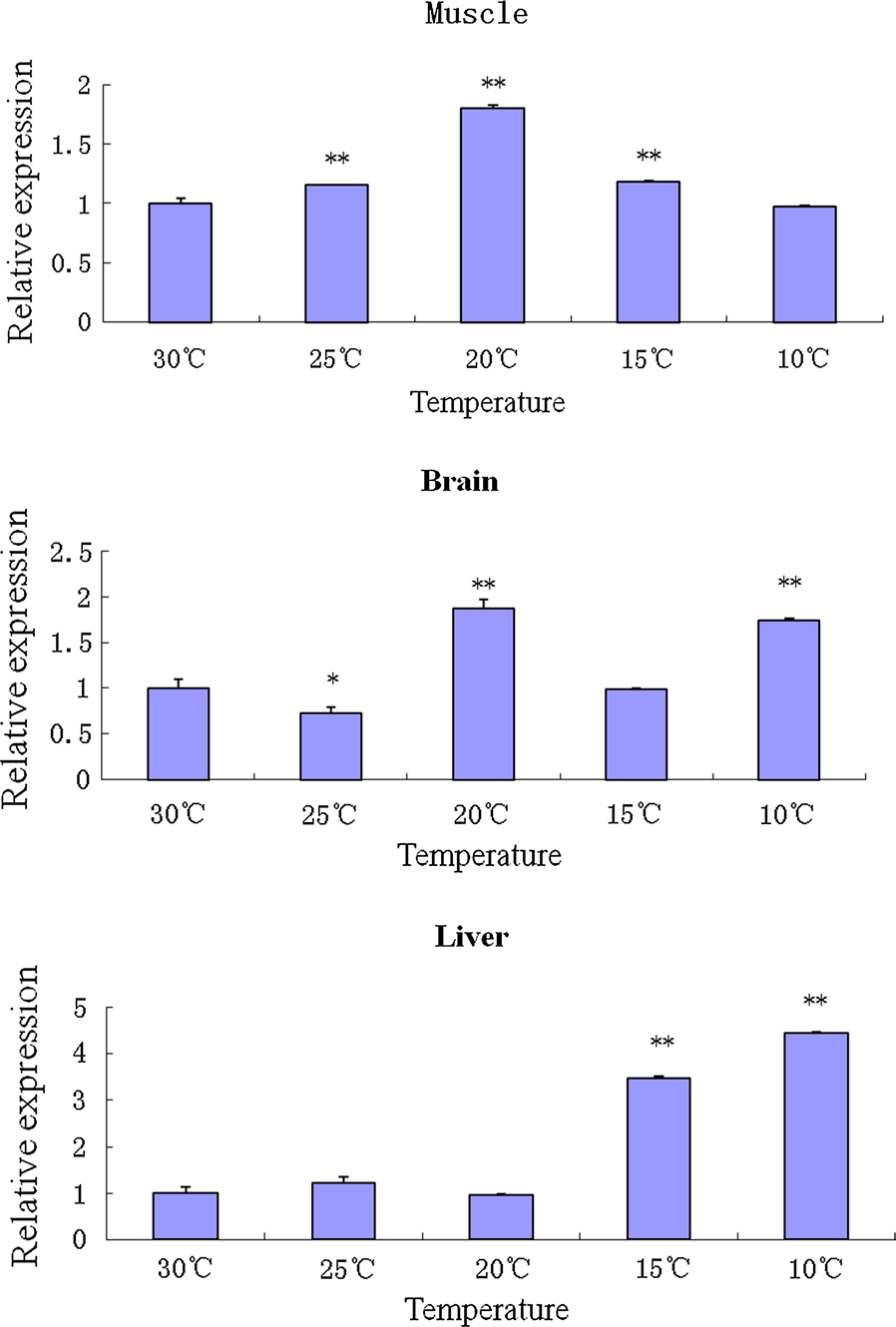
The expression pattern of tilapia *gas2* in different tissues under low temperature stress was detected by the quantitative RT-PCR. The β-actin gene was used as an internal control to calibrate the cDNA template for all the samples. Data are expressed as the mean ± SD (n = 3). Significant differences are indicated with two asterisks at *p* < 0.01, and *one asterisk* at *p* < 0.05

qRT-PCR was employed to quantify the expression of tilapia *gas2* mRNA in different tilapia tissues from normal fish and in those experiencing low-temperature stress. Tilapia *gas2* mRNA was found in all nine tissues examined, with the highest levels in the spleen and liver (Fig. 4). The *gas2* expression changed in the muscle, brain and liver when tilapia was subjected to low temperature stress. The *gas2* expression levels at 10 and 15 °C were significantly higher in the liver compared with that at 30 °C. The gas2 expression increased first and then tended to decrease in muscle from fish exposed to decreasing temperature. The *gas2* expression was significantly higher at 15, 20, and 25 °C than that at 30 °C. Similarly, *gas2* expression in the brain was significantly higher at 10 and 20 °C than that at 30 °C. In contrast, expression was significantly lower in the brain at 25 °C than at 30 °C (Fig. 5).

### Tilapia Gas2 western blot assay

**Fig. 6:**
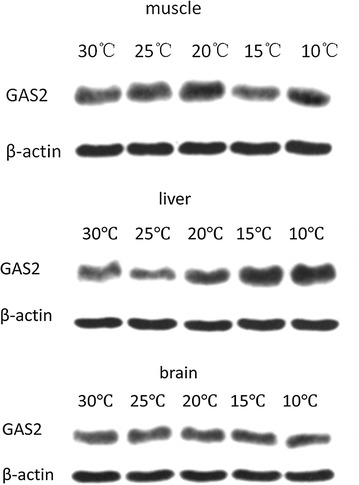
Western-blot result of tilapia Gas2 protein in four tissues under low-temperature stress

A western blot assay of Gas2 showed that Gas2 protein content in the brain did not change when temperature was decreased. Similar to *gas2* mRNA expression in muscle, Gas2 protein content was higher at 20 and 25 °C than that at 30 °C. However, Gas2 protein content in the liver was low at 30 °C and decreased as temperature was decreased, but protein content increased initially. Gas2 protein content was maximal at 10 °C (Fig. 6).

### Tilapia Gas2 subcellular localization

**Fig. 7:**
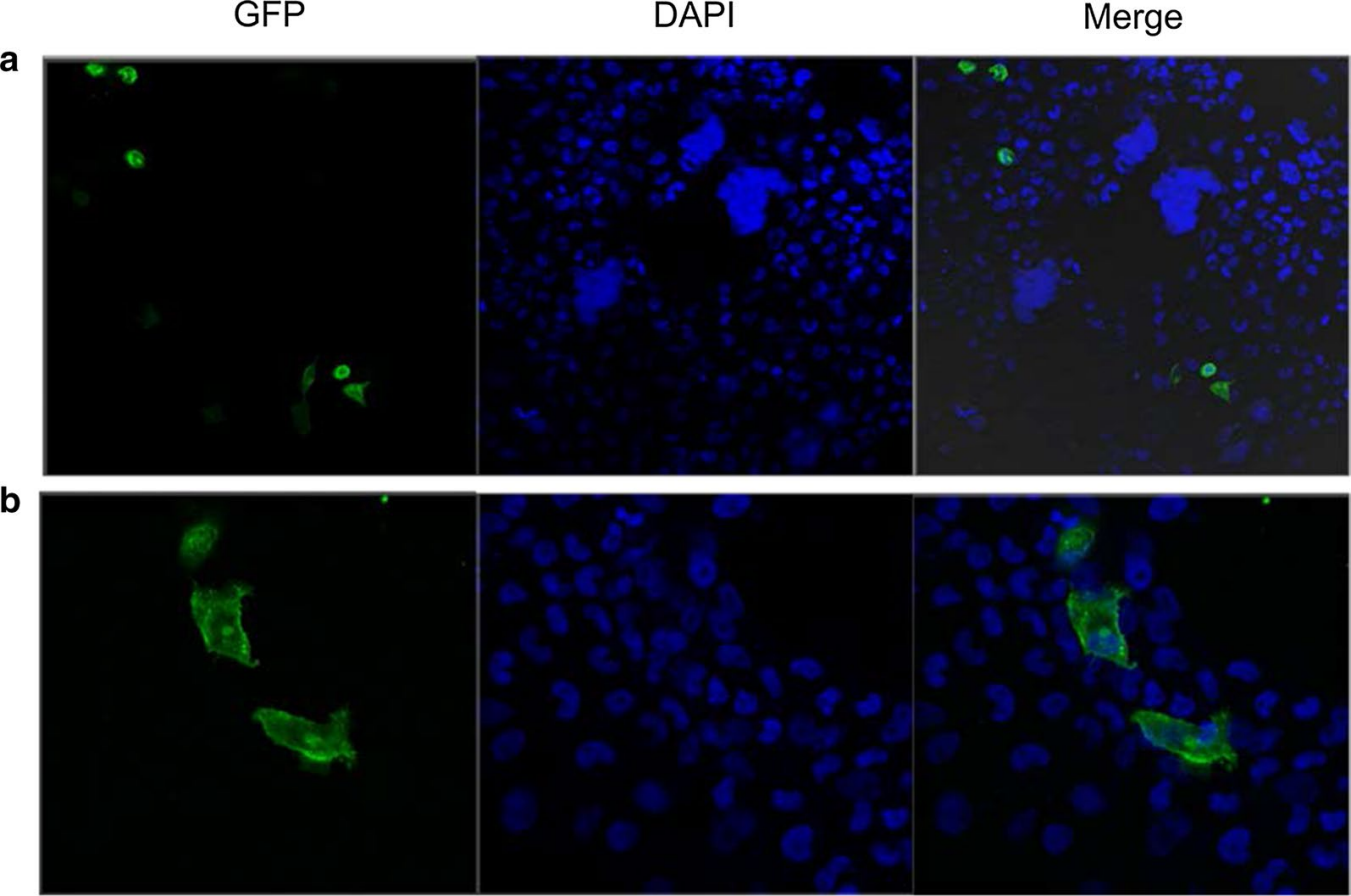
The subcellular location of tilapia Gas2 in TBC cells using a confocal laser endomicroscopy. **a** Localization of Gas2-GFP fusion green fluorescence proteins in the cytoplasm of TBC cells. **b** Control localization of GFP green fluorescence proteins

The wild-type green fluorescent protein (GFP) exhibited diffuse localization throughout the cell as seen on CLSM, whereas the transiently expressed Gas2-GFP fusion protein was distributed in a cytoplasmic network within the cell (Fig. 7).

### Tilapia *gas2* RNAi expression

**Fig. 8:**
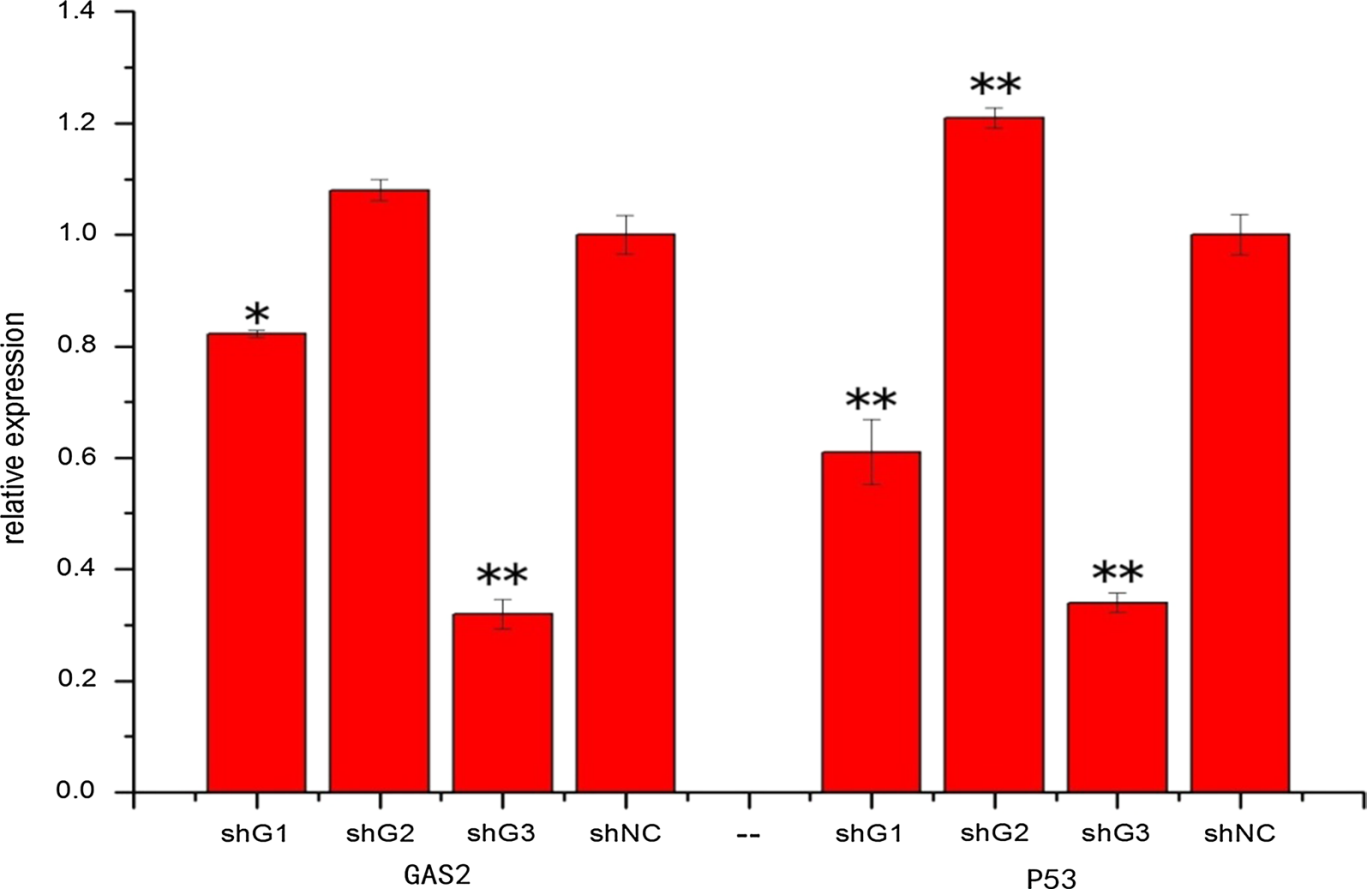
The expression pattern of *gas2* and P53 after shRNA transfection in TBC. GAS2: the relative expression of *gas2* gene after 48 h transfected shG1, shG2, shG3 and shNC. *P53* the relative expression of P53 gene after 48 h transfected shG1, shG2, shG3 and shNC. Data are expressed as the mean ± SD (n = 3). Significant differenced across medium injection were indicated with an *asterisk* at *p* < 0.05, and *two asterisk* at *p* < 0.01

After transfecting the TBC and extracting RNA, the real-time PCR results showed that shG1 and shG3 effectively reduced *gas2* expression of the transduced cells and expression decreased by 1- to 3-fold. P53 expression levels also decreased 1- to 3-fold (Fig. 8).

## Discussion

Our DGE results (unpublished data) revealed that tilapia *gas2* expression increased significantly in the liver as temperature was decreased. We cloned the tilapia *gas2* gene. The full length tilapia *gas2* cDNA and the regulatory region were also amplified by 3′- and 5′-RACE and gene walking for further study. The predicted amino acid sequence of this gene was searched against the NCBI protein database, and a comparative analysis revealed high-sequence identity of the candidate protein with Gas2 of *Maylandia zebra*. The structural features of tilapia Gas2 were consistent with the well-known CH and GAR domains. The CH domain is a family of actin binding domains found in cytoskeletal and signal transduction proteins that directly links signal transduction molecules to the actin cytoskeleton via an association with F-actin [16, 17]. Most actin-binding proteins have two copies of the CH domain, but tilapia Gas2 had a single copy. The GAR domain is common in plakin and Gas2 family members. The GAR domain comprises about 57 amino acids and binds to microtubules [18]. Tilapia Gas2 had a GAR domain containing 73 amino acids. A Gas2 domain analysis revealed that it contained the CH and GAR domains, indicating that it has the potential to bind to microtubules.

The regulatory region (–3000 to –2400 bp) exhibited relatively higher promoter activity than other promoter regions according to a functional analysis of the tilapia *gas2* 5′-flanking region. In addition, several potential transcription factor binding sites were identified in the tilapia *gas2* positive regulatory region. The transcription factors binding to these sites are related to regulation of growth and development, cell cycle regulation, cell apoptosis, and immunoregulation. For example, transcription factors, such as the Fork head box, NeuroD, SOX/SRY.

Low-temperature stress not only can lead to cell swelling and membrane damage, resulting in cell necrosis [19], but can also trigger expression of a series of genes and complex physiological responses, including inhibition of cell proliferation, cell cycle changes, and cell apoptosis [6, 20–22]. Cell apoptosis or necrosis depends largely on the intensity of the low-temperature stress. Yao et al. [23] showed that the low and high temperature stress response mechanisms are similar in mussels and are dependent on caspase-3. They suggested a causal link between DNA damage at high and low temperatures and subsequent stress reactions, such as induced cell apoptosis. Low-temperature stress affects cell membrane fluidity and cell mass transport, leading to cell division, growth arrest, and apoptosis at the cellular level in tilapia [24]. Similarly, our previous DGE study reported that multiple genes are involved in apoptosis and change as temperature decreased, such as BCL2, E3 ligase, AXIN1, and *gas2* genes [8]. The *gas2* is a multifunctional gene involved in cell apoptosis. On the one hand, the *gas2* gene is a caspase-3 substrate that is cleaved by caspase-3 and is involved in the morphological changes of apoptotic cells [13, 25], on the other overexpressing *gas2* does not directly induce apoptosis but may increase sensitivity of cells to apoptotic signals [14]. Gas2 was shown to be a component of the microfilament network system [11, 26, 27], similarly, a network of Gas2 expression was identified in the cell cytoplasm in this study. Moreover, tissue distribution of Gas2 indicated that this gene was expressed ubiquitously, with the highest expression in the liver, indicating that liver might be one of the most important locations where Gas2 carry out physiological function. In addition, the changes in tilapia *gas2* mRNA and protein levels were detected under low-temperature stress by qRT-PCR and western blot analysis. Our early DGE experimental results in tilapia liver were validated by the present results, i.e., significantly higher expression of *gas2* was detected in the liver when temperature was dropped to the lethal minimum of 10 °C than that at 30 °C. These results suggested that *gas2* synthesis was induced by low temperature, in the liver, indicating that the liver might be one of the most important tissues functioning in temperature stress response. Some studies have found that low-temperature stress can induce apoptosis of liver cells [28, 29]. Our finding of the higher expression of apoptosis-related gene (*gas2*) detected in the tilapia liver may also suggest the induction of liver cell apoptosis under low-temperature stress. The liver cells undergoing apoptosis may be related to the increasing stress sensitivity of these cells, and further results in a large effect on physiological functioning. This hypothesis may partly explain the reason for the poor low temperature tolerance of tilapia. The *gas2* RNAi results showed that shRNA was effective in reducing expression of the *gas2* gene in cells. Simultaneously, P53 gene expression also decreased. The inhibition of *gas2* gene expression may have reduced expression of the P53 gene. Some studies have shown that Gas2 affects stability of the P53 protein, and Gas2 binds to m-calpain in vivo and inhibits calpain activity, leading to increased P53 stability [14]. However, the reasons for these results are unclear and need further study.

## Conclusions

Here in this study, sequence cloning, phylogenetic analysis and functional promoter analysis were performed to better understand the characteristics of an apoptosis-related gene, tilapia *gas2*. Furthermore, qRT-PCR and western blot analyses indicated that *gas2* expression in multiple tilapia tissues (liver, muscle and brain) was significantly affected by low temperature stress. The regulated expression of this apoptosis-related gene revealed that low temperature may induce apoptosis of multiple tissues, partly explaining the sensitivity of tilapia to low-temperature on the molecular level.

## Additional files

**Additional file 1: Table S1.** Primers used in this study. The primers were used in cloning, vector construction and qRT-PCR.

**Additional file 2: Table S2.** The sequences of shRNA. The sequences information of shRNA were used in RNAi experiment.

**Additional file 3: Table S3.** List of species with their GenBank accession numbers. The species with GenBank accession numbers were used in phylogenetic analyses.

**Additional file 4: Table S4.** Phylogenetic tree of Gas2 amino acid sequences based on Neighbor-Joining (NJ) method. The bootstrap confidence values shown at the nodes of the tree are based on a 1000 bootstrap procedure, and the branch length scale in terms of genetic distance is indicated below the tree.

## Abbreviations

DGE: digital gene expression profiling
cDNA: complementary DNA
CHO-K1: Chinese hamster ovary K1 cell line
RNAi: RNA interference
DAPI: 4′,6-diamidino-2-phenylindole
IgG: immunoglobulin G
ORF: open reading frame
