# Supplementary material for: Growth arrest specific gene 2 in tilapia (Oreochromis niloticus): molecular characterization and functional analysis under low-temperature stress
Source: BMC Mol Biol. 2017 Jul 17;18:18. doi: 10.1186/s12867-017-0095-y (PMC5514492; doi:10.1186/s12867-017-0095-y)
Supplement: Supplementary file 1 — Additional file 1: Table S1. Primers used in this study. The primers were used in cloning, vector construction and qRT-PCR. [file 12867_2017_95_MOESM1_ESM.docx]

Supplementary Table 1 Primers used in this study.

| Name | Sequence 5’-3’ | Purpose used |
| --- | --- | --- |
| GAS2-5-N-A1 | AACAGTGGCTTTCCAGTCGA | 5'RACE |
| GAS2-5-W-A1 | CTCGTCCTGTGAGATGCTGGTT |  |
| GAS2-3-W-S1 | TATCCCCCATCAGCAGCAAGTC | 3'RACE |
| GAS2-3-N-S1 | GCGCACTACCGCAGCAAAAAGTG |  |
| GAS2-WL-A1 | CATTTTGCTCATATCTTATTTGTTA | gene walking |
| GAS2-WL-A2 | AACAGTGGCTTTCCAGTCGA |  |
| GAS-P-S1 | CGCGGATCCATGTGCAGTTCCCTGAGCCC | Vector construction |
| GAS-P-A1 | CCGCTCGAGCTTTTTGCTGCGGTAGTGC |  |
| GAS-N-S1 | CCCAAGCTTCGATGTGCAGTTCCCTGAGCCC |  |
| GAS-N-A1 | CGCGGATCCCTTTTTGCTGCGGTAGTGC |  |
| GAS-R-S1 | GAGATTGAGCAAGAAGAGAAAGCG | qRT-PCR |
| GAS-R-A1 | GGATGGAGAAGGAGATGAAGAGG |  |
| P53-R-S1 | GATGAACCGCAGACCTATCCTC |  |
| P53-R-A1 | GGAGGTGGAGCACTCTTTCG |  |
| Actin-S1 | TGGTGGGTATGGGTCAGAAAG |  |
| Actin-A1 | CTGTTGGCTTTGGGGTTCA |  |
