# Supplementary material for: Growth arrest specific gene 2 in tilapia (Oreochromis niloticus): molecular characterization and functional analysis under low-temperature stress
Source: BMC Mol Biol. 2017 Jul 17;18:18. doi: 10.1186/s12867-017-0095-y (PMC5514492; doi:10.1186/s12867-017-0095-y)
Supplement: Supplementary file 2 — Additional file 2: Table S2. The sequences of shRNA. The sequences information of shRNA were used in RNAi experiment. [file 12867_2017_95_MOESM2_ESM.docx]

Supplementary Table 2 the sequences of shRNA

|  |  | sequences(5'---3') |
| --- | --- | --- |
| shG1 | forward oligo nucleotide | CACCGGCCCTGTGGCTCACTAATATTTCAAGAGAATATTAGTGAGCCACAGGGCCTTTTTTG |
|  | reverse oligo nucleotide | GATCCAAAAAAGGCCCTGTGGCTCACTAATATTCTCTTGAAATATTAGTGAGCCACAGGGCC |
| shG2 | forward oligo nucleotide | CACCGCAGTAGGTGTAACAACATCATTCAAGAGATGATGTTGTTACACCTACTGCTTTTTTG |
|  | reverse oligo nucleotide | GATCCAAAAAAGCAGTAGGTGTAACAACATCATCTCTTGAATGATGTTGTTACACCTACTGC |
| shG3 | forward oligo nucleotide | CACCGCCAATGATCCACCTTGCAGATTCAAGAGATCTGCAAGGTGGATCATTGGCTTTTTTG |
|  | reverse oligo nucleotide | GATCCAAAAAAGCCAATGATCCACCTTGCAGATCTCTTGAATCTGCAAGGTGGATCATTGGC |
| shNC | forward oligo nucleotide | CACCGTTCTCCGAACGTGTCACGTCAAGAGATTACGTGACACGTTCGGAGAATTTTTTG |
|  | reverse oligo nucleotide | GATCCAAAAAAGTTCTCCGAACGTGTCACGTAATCTCTTGACGTGACACGTTCGGAGAAC |
