# Supplementary material for: Growth arrest specific gene 2 in tilapia (Oreochromis niloticus): molecular characterization and functional analysis under low-temperature stress
Source: BMC Mol Biol. 2017 Jul 17;18:18. doi: 10.1186/s12867-017-0095-y (PMC5514492; doi:10.1186/s12867-017-0095-y)
Supplement: Supplementary file 3 — Additional file 3: Table S3. List of species with their GenBank accession numbers. The species with GenBank accession numbers were used in phylogenetic analyses. [file 12867_2017_95_MOESM3_ESM.docx]

Supplementary Table 3 List of species with their GenBank accession numbers.

| Species (gene name) | Genbank accession numbers |
| --- | --- |
| *Neolamprologus brichardi* | XP_006789245 |
| *Haplochromis burtoni* | XP_014196623 |
| *Larimichthys crocea* | KKF15712 |
| *Poecilia formosa* | XP_007578649 |
| *Xiphophorus maculates* | XP_005809970 |
| *Maylandia zebra* | XP_004575167 |
| *Merops nubicus* | XP_008936602 |
| *Kryptolebias marmoratus* | XP_017261265 |
| *Fundulus heteroclitus* | XP_012735756 |
| *Homo sapiens* | NP_005247 |
| *Mus musculus* | NP_032113 |
| *Rattus norvegicus* | NP_001120976 |
| *Gallus gallus* | NP_001186399 |
| *Oryzias latipes* | XP_004069628 |
| *Macaca mulatta* | NP_001244553 |
| *Cynoglossus semilaevis* | XP_008310260 |
| *Drosophila bipectinata* | XP_017102348 |
