# Supplementary material for: Growth arrest specific gene 2 in tilapia (Oreochromis niloticus): molecular characterization and functional analysis under low-temperature stress
Source: BMC Mol Biol. 2017 Jul 17;18:18. doi: 10.1186/s12867-017-0095-y (PMC5514492; doi:10.1186/s12867-017-0095-y)
Supplement: Supplementary file 4 — Additional file 4: Table S4. Phylogenetic tree of Gas2 amino acid sequences based on Neighbor-Joining (NJ) method. The bootstrap confidence values shown at the nodes of the tree are based on a 1000 bootstrap procedure, and the branch length scale in terms of genetic distance is indicated below the tree. [file 12867_2017_95_MOESM4_ESM.docx]

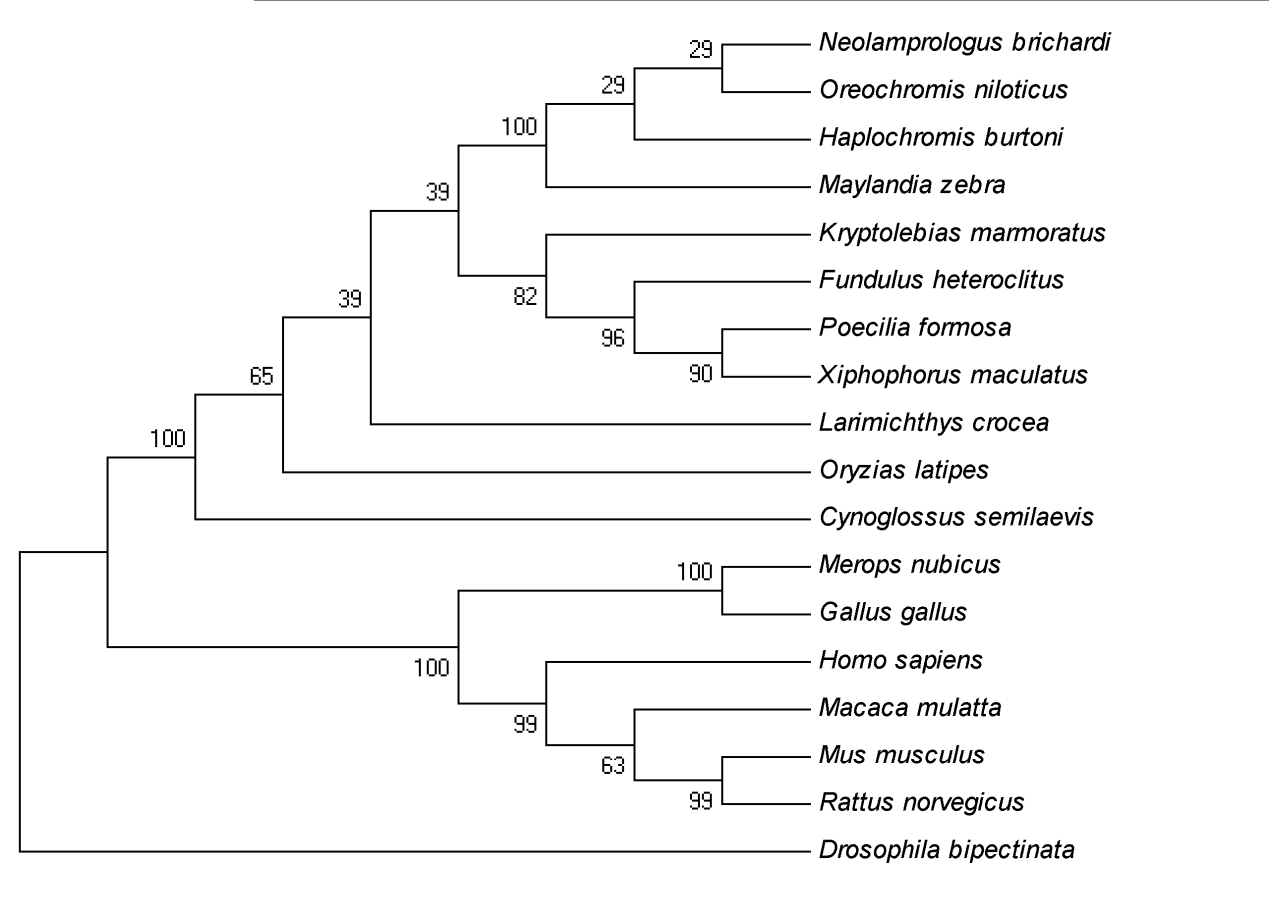


Supplementary Fig. 1. Phylogenetic tree of Gas2 amino acid sequences based on Neighbor-Joining (NJ) method. The bootstrap confidence values shown at the nodes of the tree are based on a 1000 bootstrap procedure, and the branch length scale in terms of genetic distance is indicated below the tree.
